# Hepatocyte HNF4α Deficiency Protects Against Pneumococcal Sepsis Through a Neutrophil-Dependent Mechanism

**DOI:** 10.3390/ijms27188198

**Published:** 2026-09-15

**Authors:** Jolien Vandewalle, Marah Heyerick, Steven Timmermans, Claude Libert

**Affiliations:** 1 Center for Inflammation Research, VIB, 9052 Ghent, Belgium; jolien.vandewalle@irc.vib-ugent.be (J.V.); marah.heyerick@irc.vib-ugent.be (M.H.); steven.timmermans@irc.vib-ugent.be (S.T.); 2 Department of Biomedical Molecular Biology, Ghent University, 9052 Ghent, Belgium

**Keywords:** *Streptococcus pneumoniae*, pneumonia, sepsis, liver, HNF4α, neutrophil, macrophage, metabolism, immune system, RNA-seq

## Abstract

Sepsis induces profound alterations in liver function that contribute to disease progression. We previously demonstrated that peritoneal sepsis is associated with marked disruption of hepatic transcriptional programs, including loss-of-function of Hepatocyte Nuclear Factor 4 alpha (HNF4α), a master regulator of hepatic identity and metabolism. Using hepatocyte-specific HNF4α knockout mice, we further showed that loss of HNF4α is detrimental during peritoneal sepsis. Here, we investigated whether this response is conserved in pneumonia-induced sepsis and assessed its functional significance. Bulk liver RNA sequencing revealed that suppression of hepatic metabolic pathways and HNF4α target genes are conserved features of both peritoneal and *Streptococcus pneumoniae*-induced sepsis. Unexpectedly, hepatocyte-specific HNF4α deficiency was associated with lower bacterial burden and increased survival during pneumococcal sepsis. Mechanistically, this protective effect was dependent on neutrophils and not macrophages. These findings demonstrate that sepsis-induced hepatic HNF4α dysregulation is conserved across distinct infectious etiologies but exerts context-dependent effects on disease outcome.

## 1. Introduction

Sepsis is defined as a life-threatening organ dysfunction caused by a dysregulated host response to infection. Its global burden has long been underestimated. A recent analysis estimated that sepsis contributes to 21.4 million deaths worldwide, representing 31.5% of all global mortality [1]. Despite decades of research, sepsis remains a major unmet medical need, in part because the underlying pathophysiology remains incompletely understood and disease-modifying therapies are thus lacking.

Susceptibility to sepsis is determined by both pathogen-related factors (virulence, inoculum, infection site) and host characteristics (age, comorbidities, immune status). The infectious insult triggers a dysregulated host response that simultaneously activates and suppresses multiple physiological systems. Among affected organs, the liver plays a central role in coordinating metabolic, inflammatory, and immunological responses to infection [2,3]. As a key regulator of systemic homeostasis, the liver integrates signals from pathogens, cytokines, and metabolic stressors to orchestrate the acute-phase response and maintain energy balance. During severe infection, however, this regulatory capacity becomes compromised, leading to widespread metabolic dysfunction that contributes to organ failure and poor clinical outcomes [4,5].

A recently introduced theory is that sepsis induces a maladaptive starvation response [2]. Reduced food intake, combined with enhanced lipolysis in white adipose tissue (WAT), results in the release of free fatty acids (FFAs) intended to fuel hepatic β-oxidation [6]. Yet, during cecal ligation and puncture (CLP)-induced peritoneal sepsis, β-oxidation pathways are suppressed [6]. Hepatocyte Nuclear Factor 4 alpha (HNF4α), a master regulator of liver-specific gene expression, has recently been identified as a key driver of this metabolic collapse [7]. As evidenced by inducible hepatocyte-specific HNF4α deletion (HNF4α^Liver-i-KO^) mice, loss of HNF4α activity following peritoneal sepsis impairs PPARα-dependent β-oxidation, promotes FFA accumulation, disrupts the acute-phase response, and ultimately induces lethality [7]. Conversely, both prophylactic and therapeutic interventions that stimulate HNF4α have been shown to protect against peritoneal sepsis by restoring these metabolic and inflammatory responses [7].

Although hepatic metabolic dysfunction has been well documented in peritoneal sepsis and across multiple species, including mice [6,7,8], dogs [9], and pigs [8,10], it remains unclear whether similar hepatic disturbances occur during respiratory infections that progress to sepsis. This question is particularly relevant because the respiratory tract is the most common source of sepsis in human patients (±67.4%), followed by abdominal (±21.8%) and urogenital (±15.6%) infections [11]. In contrast, most preclinical studies rely on CLP- induced peritoneal sepsis (±51%) or LPS-induced sterile inflammation (~48%), the latter actually does not fully recapitulate human sepsis [12]. Only a minority of studies use pneumonia-induced sepsis models (±7%), despite pneumonia being the dominant cause of sepsis in humans [12]. Addressing this knowledge gap is important to determine whether the hepatic metabolic response and the role of HNF4α described in peritoneal sepsis extend to pneumonia-induced sepsis, thereby assessing the generalizability of these findings beyond the peritoneal sepsis model and the broader therapeutic relevance of HNF4α stimulation in sepsis.

To address this question, we performed a comprehensive analysis of the liver transcriptome in mice infected intratracheally with *Streptococcus pneumoniae*. We further examined the functional consequences of HNF4α loss-of-function following pneumococcal infection using HNF4α^Liver-i-KO^ mice. *S. pneumoniae* was chosen as the inoculum because it is the most common cause of community-acquired pneumonia worldwide [13].

## 2. Results

### 2.1. Hepatic Metabolic Suppression and HNF4α Loss During S. pneumoniae Infection

**Figure 1:**
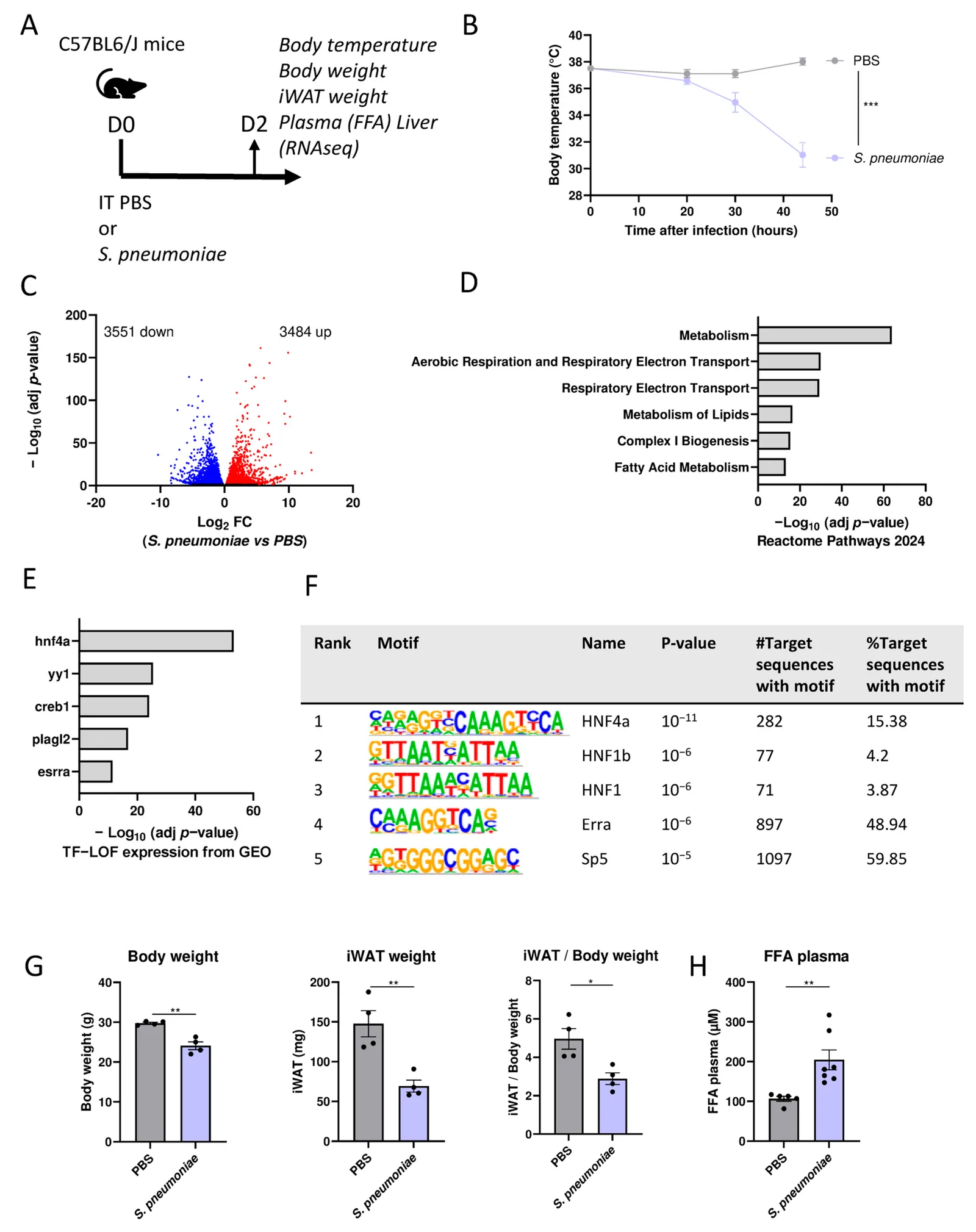
Hepatic metabolic suppression and HNF4α loss during *S. pneumoniae* infection. (**A**) Experimental setup. (**B**) Rectal body temperature was monitored for two days following intratracheal (IT) instillation of *S. pneumoniae* (n = 3) or PBS (n = 3/group). (**C**) Volcano plot showing differentially expressed genes in the liver two days after *S. pneumoniae* infection compared with PBS (n = 3/group). Genes significantly upregulated following *S. pneumoniae* infection are indicated in red, whereas significantly downregulated genes are indicated in blue (*p* < 0.05). (**D**) Top six enriched Reactome pathways identified using Enrichr among genes downregulated in the liver following *S. pneumoniae* infection (*p* < 0.05). (**E**) Top five enriched transcription factor loss-of-function (TF-LOF) signatures from GEO (Enrichr) among genes downregulated following *S. pneumoniae* infection (*p* < 0.05). (**F**) HOMER motif analysis of DNA-binding motifs within the 500 bp upstream of the transcription start sites of genes downregulated following *S. pneumoniae* infection. The top five motifs ranked by *p*-value are shown. (**G**) Body weight, inguinal white adipose tissue (iWAT) weight, iWAT-to-body weight ratio (n = 4/group), and (**H**) plasma-free fatty acid (FFA) levels (n = 5 for PBS and n = 7 for *S. pneumoniae*) were determined two days following *S. pneumoniae* or PBS instillation. *p*-values in panel (**B**) were determined using a mixed-effects model with time and treatment as fixed effects and individual animals as the repeated measure. *p*-values in panels (**G**,**H**) were determined using an unpaired *t*-test. ns, *p* ≥ 0.05; *, *p* < 0.05; **, *p* < 0.01; ***, *p* < 0.001.

Substantial reprogramming of liver metabolic pathways is a hallmark of peritoneal sepsis [2,7]. To determine whether these metabolic disturbances are conserved across different sepsis models, we performed functional analysis of bulk liver RNA-seq data collected two days after intratracheal *S. pneumoniae* infection with a lethal dose, corresponding to the time point at which mice begin to succumb to infection, as reflected by a drop in body temperature (Figure 1A,B). Compared to mock-infected controls (PBS), 7035 genes were differentially expressed in the liver (*p*adj < 0.05), with 3551 genes being downregulated (blue dots) and 3484 upregulated (red dots) following *S. pneumoniae* infection (Figure 1C).

Because the shutdown of usual cellular activities is central to sepsis pathology [14], we focused specifically on genes that were downregulated after infection. Gene ontology enrichment revealed ‘Metabolism’ as the most significantly affected category, followed by more specific pathways such as ‘respiratory electron transport’ and ‘metabolism of lipids’ (Figure 1D). Notably, suppression of ‘lipid metabolism’ and ‘fatty acid metabolism’ closely mirrors the transcriptional changes observed in the liver during peritoneal sepsis (CLP model) [6,7]. This convergence indicates that dysregulation of these metabolic pathways is a conserved feature of sepsis, independent of the infectious insult or experimental model.

Transcription factor loss-of-function analysis further revealed that many of these downregulated genes (e.g., *Ppara*) are controlled by HNF4α (Figure 1E), a key hepatic nuclear factor that also progressively loses activity during peritoneal sepsis [7]. Consistently, HOMER motif analysis of all downregulated genes showed strong enrichment of HNF4α binding motifs in their promoter regions (Figure 1F). HNF4α is a nuclear receptor essential for regulating lipid metabolism, fatty-acid oxidation, and overall energy homeostasis, making its loss-of-function a plausible driver of the observed metabolic collapse.

A maladapted starvation response has been implicated in sepsis pathophysiology [2]. This response is characterized by reduced food intake followed by enhanced lipolysis in white adipose tissue (WAT), releasing high-energy metabolites such as free fatty acids (FFAs) to fuel β-oxidation [6]. However, due to the loss of key transcription factors involved in β-oxidation, particularly PPARα and its downstream targets, FFAs accumulate in the plasma instead of being efficiently metabolized [6]. To determine whether increased lipolysis and FFA accumulation also occur during *S. pneumoniae* infection, we measured body weight, iWAT weight, iWAT/body weight ratio, and plasma FFA levels (Figure 1G,H). Similarly as observed in peritoneal sepsis [6], *S. pneumoniae* infection resulted in reduced body weight, iWAT weight, iWAT/body weight ratio, and elevated plasma FFAs.

Taken together, these findings demonstrate that hepatic metabolic suppression and HNF4α loss-of-function are conserved features of sepsis, independent of the infectious insult initiating the disease.

### 2.2. Hepatocyte-Specific HNF4α Loss Protects Against S. pneumoniae Infection

**Figure 2:**
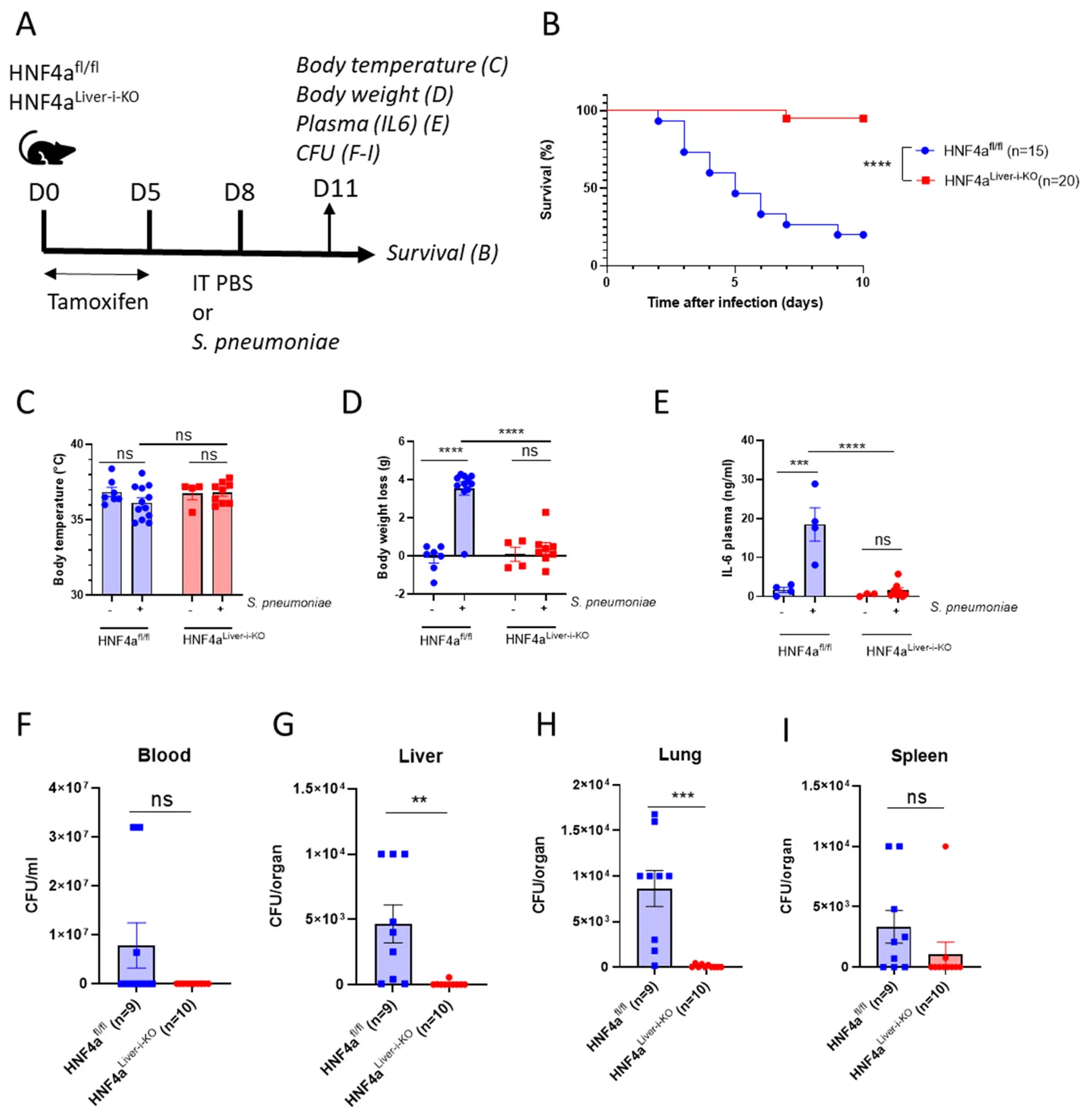
Hepatocyte-specific HNF4α loss protects against *S. pneumoniae* infection. (**A**) Experimental setup. (**B**) Survival was monitored over ten days post *S. pneumoniae* infection in HNF4α^fl/fl^ (control, n = 15) or HNF4α^Liver-i-KO^ mice (n = 20). (**C**) Body temperature, (**D**) body weight and (**E**) plasma IL-6 levels determined three days post *S. pneumoniae* or PBS infection in HNF4α^fl/fl^ (control) or HNF4α^Liver-i-KO^ mice ((**C**,**D**) n = 6 HNF4α^fl/fl^ + PBS; n = 12 HNF4α^fl/fl^ + *S. pneumoniae*; n = 4 HNF4α^Liver-i-KO^ + PBS; n = 9 HNF4α^Liver-i-KO^ + *S. pneumoniae*; (**E**) n = 4 HNF4α^fl/fl^ + PBS; n = 4 HNF4α^fl/fl^ + *S. pneumoniae*; n = 3 HNF4α^Liver-i-KO^ + PBS; n = 8 HNF4α^Liver-i-KO^ + *S. pneumoniae*). (**F**–**I**) Colony-forming units (CFU) were determined three days post *S. pneumoniae* infection in HNF4α^fl/fl^ (control, n = 9) or HNF4α^Liver-i-KO^ mice (n = 10) in blood (**F**), liver (**G**), lung (**H**) and spleen (**I**). *p*-value for panel (**B**) was analyzed with a Log-rank test. *p*-values for panels (**C**–**E**) were analyzed with two-way ANOVA. *p*-values for panels (**F**–**I**) were analyzed with an unpaired *t*-test. ns = *p* ≥ 0.05; ** = *p* < 0.01; *** = *p* < 0.001; **** = *p* < 0.0001.

To investigate the impact of hepatic HNF4α loss-of-function on pneumonia-induced sepsis progression, we evaluated *S. pneumoniae* infection outcomes in HNF4α^Liver-i-KO^ mice (Figure 2A). In striking contrast to their heightened susceptibility in the peritoneal sepsis (CLP) model [7], HNF4α^Liver-i-KO^ mice were almost completely protected against intratracheally initiated pneumococcal infection, exhibiting 95% survival compared with only 20% in control littermates (Figure 2B).

By 3 days post-infection, WT controls began to develop hypothermia (Figure 2C), but their clinical deterioration was most evident from body weight loss. WT mice lost approximately 4 g within this short time frame (Figure 2D), whereas HNF4α^Liver-i-KO^ mice maintained both body temperature and body weight, showing no overt signs of illness.

To assess systemic inflammation, we measured plasma IL-6 levels in both genotypes at baseline and after infection. Consistent with their clinical decline, WT mice displayed a marked increase in IL-6 following *S. pneumoniae* challenge (Figure 2E). In contrast, IL-6 levels in HNF4α^Liver-i-KO^ mice remained nearly unchanged, mirroring their preserved physiological state. Because elevated IL-6 has been associated with a higher bacterial load [15], we next examined whether reduced bacterial burden might underlie the protection observed in HNF4α^Liver-i-KO^ mice. Bacterial counts were quantified in blood, liver, lung, and spleen (Figure 2F–I). Across all tested tissues, HNF4α^Liver-i-KO^ mice exhibited lower or even undetectable bacterial loads, with significant reductions in both liver and lung. These findings suggest that the protection conferred by hepatic HNF4α loss-of-function is associated with improved bacterial control during *S. pneumoniae* infection.

### 2.3. KCs Are Dispensable for HNF4α^Liver-i-KO^ Protection

**Figure 3:**
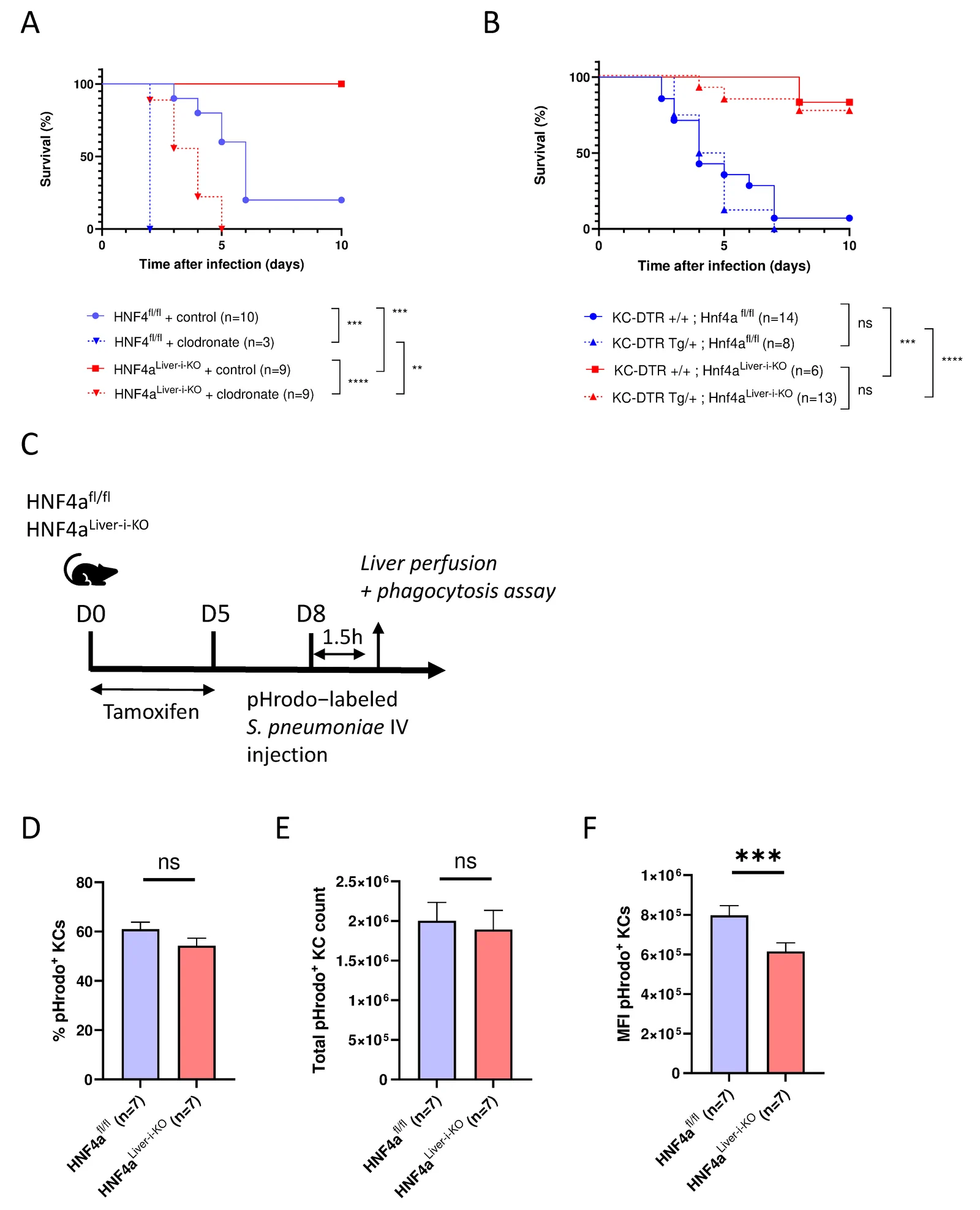
Kupffer cells (KCs) are dispensable for HNF4α^Liver-i-KO^ protection. (**A**) HNF4α^fl/fl^ (control) or HNF4α^Liver-i-KO^ mice were injected intravenously with clodronate or control liposomes one day before infection with *S. pneumoniae*. Survival was then followed over ten days post *S. pneumoniae* infection. The number of animals (n) per group is indicated in the figure. (**B**) Double-transgenic KC-DTR^Tg/+^; HNF4α^Liver-i-KO^ mice and their respective controls were subjected to *S. pneumoniae* infection following diphtheria toxin (DT) treatment. Survival was monitored over ten days post *S. pneumoniae* infection. The number of animals (n) per group is indicated in the figure. (**C**) Experimental setup for (**D**–**F**). (**D**) Percentage of pHrodo-positive KCs, (**E**) absolute number of pHrodo-positive (phagocytosing) KCs per liver and (**F**) mean fluorescence intensity (MFI) of pHrodo-positive KCs among HNF4α^fl/fl^ (control, n = 7) or HNF4α^Liver-i-KO^ (n = 7) mice 1.5 h post pHrodo-labeled *S. pneumoniae* injection intravenously (IV). *p*-values for panels (**A**,**B**) were analyzed with a Log-rank test. *p*-values for panels (**D**–**F**) were analyzed with a generalized linear model (GLM) in RStudio. Plots show adjusted means ± SD. ns = *p* ≥ 0.05; ** = *p* < 0.01; *** = *p* < 0.001; **** = *p* < 0.0001.

To further dissect the mechanism underlying the improved bacterial control in HNF4α^Liver-i-KO^ mice following *S. pneumoniae* infection, we hypothesized that macrophages, key mediators of bacterial phagocytosis, might exhibit altered activity in these mice. To test the contribution of macrophages to the protective phenotype, HNF4α^Liver-i-KO^ and control mice were treated intravenously with clodronate liposomes one day prior to infection. Clodronate pretreatment markedly sensitized HNF4α^Liver-i-KO^ mice, reducing survival from 100% in control-liposome-treated animals to 0% in clodronate-treated mice (Figure 3A). WT mice treated with control liposomes were substantially more susceptible to *S. pneumoniae* infection than HNF4α^Liver-i-KO^ mice with control liposomes, and clodronate treatment further exacerbated the vulnerability in WT mice, decreasing survival from 20% to 0%.

Because clodronate can have off-target effects beyond macrophage depletion, we next employed a genetic approach to specifically ablate the Kupffer cells (KCs), which are liver-resident macrophages. Given that HNF4α deletion is restricted to hepatocytes, we reasoned that KCs would be the most relevant macrophage population to examine. To this end, HNF4α^Liver-i-KO^ mice were crossed with KC-DTR mice, in which KCs express the human diphtheria toxin receptor under the *Clec4f* promoter. Administration of diphtheria toxin (DT) has been shown to induce near-complete KC depletion [16] and, via RT-qPCR, we could also verify KC loss in our model, as *Clec4f* expression was nearly undetectable in KC-DTR^Tg/+^ mice (Figure S1A).

Double-transgenic KC-DTR^Tg/+^; HNF4α^Liver-i-KO^ mice and their respective controls were then subjected to *S. pneumoniae* infection following DT treatment. In contrast to the clodronate experiments, genetic KC depletion did not sensitize HNF4α^Liver-i-KO^ mice, nor did it further sensitize WT controls (Figure 3B). The protective phenotype of HNF4α^Liver-i-KO^ mice remained fully intact in both KC-sufficient and KC-depleted conditions.

Because genetic KC depletion targets only liver-resident macrophages, we reasoned that other macrophage populations might compensate for KC loss, masking any survival differences. To more directly assess KC function, we performed a phagocytosis assay on isolated KCs from HNF4α^Liver-i-KO^ and control mice. Mice were injected intravenously with pHrodo-labeled *S. pneumoniae* to induce systemic infection, and KCs were isolated 1.5 h later using Cytek sorting (Figure 3C). Although absolute KC numbers were unchanged (Figure S2A), the relative proportion of KCs among CD45^+^ cells was significantly reduced in HNF4α^Liver-i-KO^ mice (Figure S2B), indicating increased infiltration of other immune cells into the liver. Importantly, both the absolute and relative numbers of pHrodo^+^ KCs were comparable between both genotypes (Figure 3D,E). However, the mean fluorescence intensity (MFI) of pHrodo^+^ KCs was significantly lower in HNF4α^Liver-i-KO^ mice (25% reduction), suggesting a reduced bacterial uptake per KC (Figure 3F). These findings do not support a role for KCs in mediating the enhanced protection observed in HNF4α^Liver-i-KO^ mice.

Taken together, these results demonstrate that the enhanced resistance of HNF4α^Liver-i-KO^ mice to *S. pneumoniae* infection is not dependent on KCs, and that the clodronate-mediated sensitization likely reflects depletion of other macrophage populations or off-target effects. Indeed, recent work has shown that clodronate liposomes are also ingested by neutrophils, leading to their functional impairment [17]. Even more striking, the former study suggests that the effects of clodronate liposomes may be attributable primarily to neutrophil depletion rather than macrophage loss [17].

### 2.4. Neutrophils Are Required for HNF4α^Liver-i-KO^ Protection

**Figure 4:**
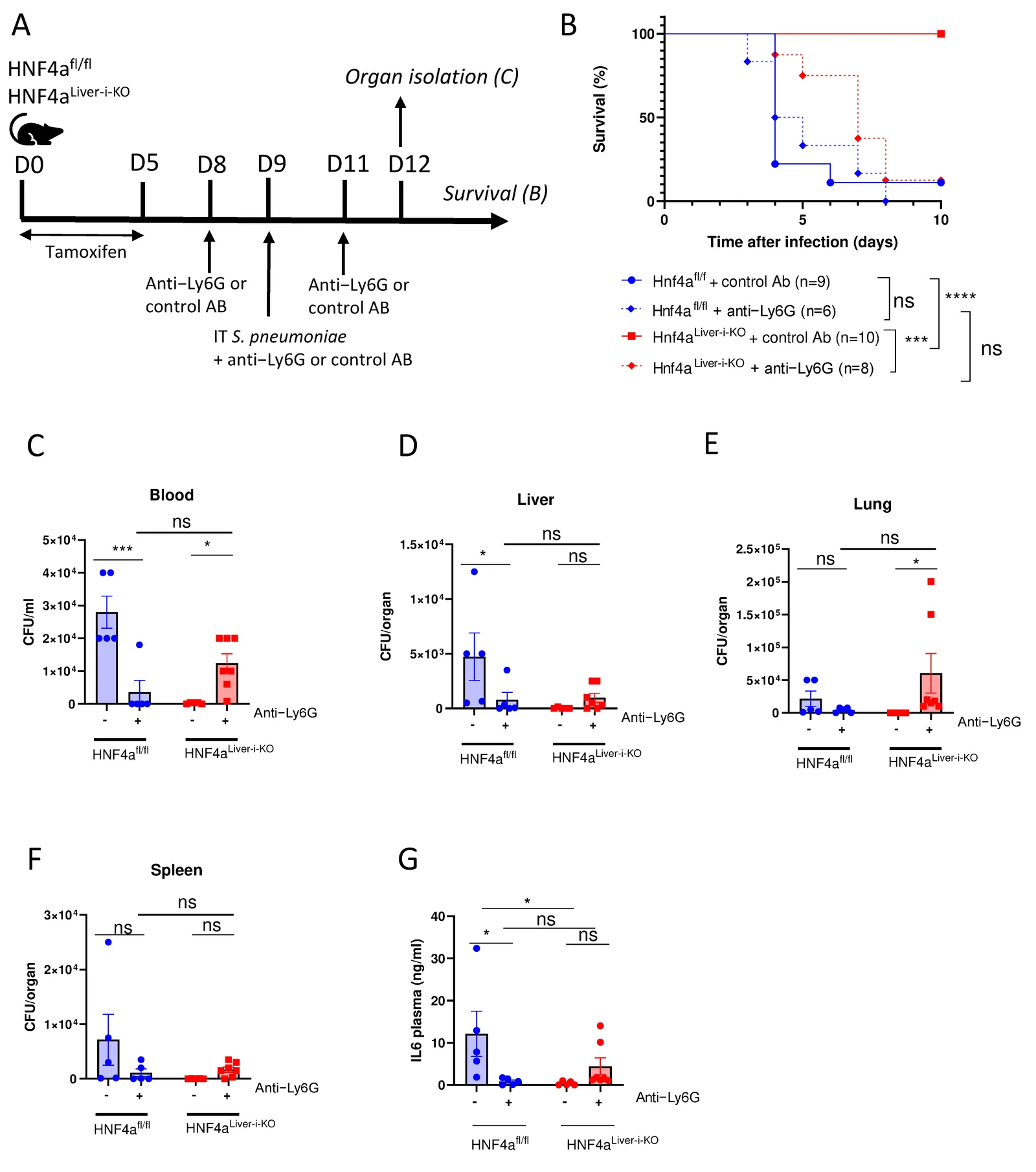
Neutrophils are required for HNF4α^Liver-i-KO ^protection. (**A**) Experimental setup. HNF4α^fl/fl^ (control) and HNF4α^Liver-i-KO^ mice were injected intraperitoneally with anti-Ly6G or control antibody on days −1, 0, and 2 relative to *S. pneumoniae* infection. (**B**) Survival was monitored for ten days following infection. The number of animals (n) per group is indicated in the figure. (**C**–**F**) Colony-forming units (CFUs) in blood (**C**), liver (**D**), lung (**E**), and spleen (**F**) and plasma IL-6 levels (**G**) were determined three days following *S. pneumoniae* infection in HNF4α^fl/fl^ (control) or HNF4α^Liver-i-KO^ mice. n = 5 HNF4α^fl/fl^ + control Ab; n = 5 HNF4α^fl/fl^ + anti-Ly6G; n = 5 HNF4α^Liver-i-KO^ + control Ab; n = 7 HNF4α^Liver-i-KO^ + *anti-Ly6G. p*-values for panel (**B**) were determined using a log-rank test. *p*-values for panels (**C**–**G**) were determined using two-way ANOVA. ns, *p* ≥ 0.05; *, *p* < 0.05; ***, *p* < 0.001; ****, *p* < 0.0001.

To dissect the role of neutrophils in mediating the protection observed in HNF4α^Liver-i-KO^ mice, we pretreated HNF4α^Liver-i-KO^ and control mice with anti-Ly6G, an antibody used to deplete neutrophils, one day prior to infection. Additional doses of anti-Ly6G were administered on the day of infection and two days post-infection to sustain neutrophil depletion throughout the experimental period (Figure 4A). Anti-Ly6G treatment markedly reduced the survival of HNF4α^Liver-i-KO^ mice from 100% to 13%, approaching that of WT mice + control antibody (11%). In contrast, anti-Ly6G treatment did not significantly alter survival in WT mice, which remained low (0% survival) (Figure 4B). These findings are in contrast with the clodronate treatment described above, which reduced survival in both WT and HNF4α^Liver-i-KO^ mice but did not completely abolish the survival advantage of HNF4α^Liver-i-KO^ mice. Thus, anti-Ly6G treatment completely abrogated the genotype-dependent survival advantage observed in HNF4α^Liver-i-KO^ mice, supporting a critical role for neutrophils in the protective phenotype of HNF4α^Liver-i-KO^ mice. The loss of protection was accompanied by a significantly increased bacterial burden in the blood and lungs, with a similar trend observed in the liver and spleen (Figure 4C–F). Remarkably, anti-Ly6G treatment had the opposite effect in WT mice, significantly reducing bacterial loads in the blood and liver. Similar trends were observed when measuring plasma IL-6 levels in these mice. As shown previously, IL-6 plasma levels were significantly lower in HNF4α^Liver-i-KO^ mice following *S. pneumoniae* infection. Anti-Ly6G treatment, however, significantly reduced IL-6 levels in WT mice, whereas an increasing trend was observed in HNF4α^Liver-i-KO mice^ (Figure 4G).

To further investigate how hepatocyte-specific HNF4α loss of function might impact neutrophil activity, we hypothesized that systemic alterations in HNF4α^Liver-i-KO^ mice could modulate neutrophil function. To explore potential explanations for the altered neutrophil phenotype, we analyzed a publicly available bulk RNA-seq dataset (GSE260635, ref. [7]) from livers of HNF4α^Liver-i-KO^ mice and their respective WT controls (Figure S3A). Among the upregulated genes in HNF4α^Liver-i-KO^ livers, the top Reactome pathways were all related to translation (initiation, elongation, ribosome assembly), indicating increased global protein synthesis (Figure S3B). This is consistent with the loss of hepatocyte identity and the activation of regenerative and proliferative programs that occur upon HNF4α depletion. Among the downregulated genes, the top pathway was ‘cholesterol biosynthesis’ (Figure S3C). When examining the expression of enzymes involved in cholesterol biosynthesis, nearly all genes were significantly reduced in livers of HNF4α^Liver-i-KO^ mice in steady-state (Figure S3D). This transcriptional repression was associated with a profound decrease in plasma total cholesterol, with basal levels in HNF4α^Liver-i-KO^ mice being approximately 14-fold lower than in WT animals, approaching near-undetectable values (Figure S3E). Cholesterol availability has been implicated in the regulation of neutrophil activation and function [18,19], raising the possibility that altered systemic cholesterol homeostasis in HNF4α^Liver-i-KO^ mice may modify the neutrophil response to pneumococcal infection. In this context, neutrophils may adopt a functionally distinct phenotype in HNF4α^Liver-i-KO^ mice, rendering these animals more dependent on neutrophils for effective antibacterial defense. This hypothesis could potentially explain why neutrophil depletion markedly increased bacterial burden and mortality in HNF4α^Liver-i-KO^ mice, whereas it did not affect survival in WT animals. Further studies are warranted to validate the hypothesis that the hypocholesterolemic phenotype of HNF4α^Liver-i-KO^ mice contributes to the altered role of neutrophils during pneumococcal infection.

## 3. Discussion

Sepsis is characterized by profound alterations in hepatic metabolism, yet the contribution of these metabolic changes to disease outcome remains incompletely understood. Our research group previously demonstrated that polymicrobial sepsis induces a progressive loss of HNF4α activity in the liver, a master regulator of hepatocyte identity and metabolic homeostasis [7]. In the present study, we extend these findings by showing that hepatic metabolic suppression and HNF4α loss-of-function are also prominent features of pneumonia-induced sepsis. Despite this shared transcriptional response, however, the functional consequences of HNF4α loss differed strikingly between the two sepsis models. Whereas hepatocyte-specific HNF4α deficiency increased susceptibility to CLP-induced sepsis [7], HNF4α^Liver-i-KO^ mice were strongly protected against *S. pneumoniae* infection. These findings indicate that HNF4α loss-of-function is a conserved component of the hepatic response to sepsis, but that its physiological consequences are highly dependent on the infectious context.

The increased susceptibility of HNF4α^Liver-i-KO^ mice in the CLP model is consistent with the essential role of HNF4α in maintaining hepatic metabolic function, the acute-phase response, and liver regenerative capacity [7]. However, bacterial burden was not assessed in the former study, limiting our ability to determine whether the increased susceptibility was primarily related to impaired host defence or to compromised hepatic metabolic and regenerative functions. In contrast, in the pneumonia-induced sepsis model, HNF4α^Liver-i-KO^ mice exhibited a reduced bacterial burden together with sustained plasma IL-6 levels, body temperature, and body weight. These findings suggest that, in the context of *S. pneumoniae* infection, HNF4α loss may promote a host response that enhances bacterial control and preserves systemic physiological function. Thus, although hepatic metabolic suppression is observed in both models, its downstream consequences may depend on the nature and/or localization of the infectious insult. Whether this context dependency is determined by the site of infection (lung versus peritoneal cavity) or by the nature of the infectious insult (monomicrobial versus polymicrobial) remains unclear. Further studies using HNF4α^Liver-i-KO^ mice in additional models of sepsis, including monomicrobial and polymicrobial infections at different anatomical sites, could help distinguish between these possibilities and clarify the determinants of the divergent effects of HNF4α loss-of-function. Nevertheless, these findings emphasize the importance of evaluating potential therapeutic strategies across different experimental models of sepsis, as the same intervention can result in markedly different, or even opposing, outcomes depending on the context.

Strikingly, anti-Ly6G-mediated neutrophil depletion completely abolished the protection conferred by HNF4α deficiency and resulted in a substantial increase in bacterial burden. These findings establish neutrophils as essential mediators of the protective phenotype. Remarkably, neutrophil depletion had markedly different effects in WT mice. In contrast to HNF4α^Liver-i-KO^ mice, anti-Ly6G reduced bacterial loads in WT mice. Previous studies have shown that neutrophils are required for protection during the early stages of *S. pneumoniae* infection, but can become detrimental at later time points [20]. Indeed, anti-Ly6G administration prior to TIGR4 instillation increases mortality and is associated with a higher bacterial burden, whereas anti-Ly6G administration 18 h after the infection decreases *S. pneumoniae*-induced mortality and is associated with a lower bacterial burden [20]. In the current study, anti-Ly6G was administered one day before infection and twice after infection to achieve sustained neutrophil depletion, as anti-Ly6G-mediated depletion is known to be transient due to rapid neutrophil replenishment from the bone marrow [21]. The reduced bacterial burden following anti-Ly6G treatment in WT mice may therefore primarily reflect the effects of neutrophil depletion during the later stages of infection, when neutrophils may exert detrimental effects [20]. Conversely, the absence of a survival benefit despite the reduced bacterial burden in WT mice may reflect the opposing, time-dependent roles of neutrophils during pneumococcal infection, whereby their early protective effects are counterbalanced by detrimental effects at later stages. It would also be of interest to assess bacterial loads at later time points, particularly around the onset of clinical deterioration in WT mice, to determine whether bacterial burden increases during this critical stage of infection and whether this is differentially affected by neutrophil depletion.

The mechanism by which hepatocyte-specific HNF4α deficiency alters neutrophil dependency remains unclear. Because HNF4α deletion is driven by the hepatocyte-specific Alb-Cre system, a direct effect of HNF4α loss in neutrophils is unlikely. Rather, our findings suggest that changes in the systemic metabolic environment may influence neutrophil behaviour. In support of this idea, analysis of liver transcriptomes from HNF4α^Liver-i-KO^ mice revealed profound suppression of cholesterol biosynthesis pathways, which was associated with an approximately 14-fold reduction in circulating total cholesterol levels. Emerging evidence suggests that cholesterol metabolism influences several aspects of neutrophil biology, including activation, migration, antimicrobial responses, and extracellular trap formation [18,19]. For example, pharmacological inhibition of cholesterol biosynthesis with statins has been shown to modulate neutrophil antibacterial functions, including enhancing phagocyte extracellular trap formation and bacterial killing, while affecting other effector functions such as phagocytosis and oxidative burst [19]. These findings indicate that alterations in cholesterol biosynthesis can directly influence neutrophil functional responses during bacterial infection. We therefore speculate that the profound hypocholesterolemic state observed in HNF4α^Liver-i-KO^ mice may contribute to the altered neutrophil dependency during pneumococcal infection. This hypothesis, however, remains to be tested. We attempted to restore plasma cholesterol levels in HNF4α^Liver-i-KO^ mice to determine whether normalization of cholesterol availability would abrogate their protective phenotype, but were unable to achieve cholesterol levels comparable to those of WT mice. Consequently, the causal contribution of hypocholesterolemia could not be directly assessed. Future studies could address this question by determining cholesterol levels in isolated neutrophils, particularly membrane-associated cholesterol, and by comparing WT and HNF4α^Liver-i-KO^ neutrophils ex vivo. Assessment of bacterial killing, phagocytosis, reactive oxygen species production, and neutrophil extracellular trap formation may help establish whether altered cholesterol availability is associated with functional changes in neutrophils derived from HNF4α^Liver-i-KO^ mice.

More broadly, our findings support a model in which hepatocyte-specific HNF4α loss alters the systemic metabolic environment, with secondary effects on neutrophil function and the host response to pneumococcal infection. Cholesterol metabolism represents one potential mediator, but the effects may extend beyond circulating cholesterol itself. Other metabolic pathways altered by HNF4α deficiency, including the mevalonate pathway, bile acid metabolism, and LXR/RXR- and PPARα-dependent signalling, might also influence neutrophil behaviour [22]. Future studies should therefore determine whether the altered neutrophil phenotype reflects a specific consequence of hypocholesterolemia or a broader metabolic reprogramming induced by hepatic HNF4α loss. Such studies may help define how hepatic metabolic alterations are translated into changes in neutrophil recruitment, activation, and antimicrobial activity during pneumococcal infection.

From a translational perspective, our findings highlight the context-dependent effects of targeting hepatic HNF4α in sepsis. While HNF4α stimulation with NCT was shown to be beneficial in polymicrobial peritonitis [7], HNF4α loss was protective in pneumococcal pneumonia, emphasizing that therapeutic strategies may not have uniform effects across sepsis contexts. The availability of experimental HNF4α antagonists, such as BI6015, provides an opportunity to test whether pharmacological HNF4α inhibition can reproduce the protective phenotype observed in HNF4α^Liver-i-KO^ mice during pneumonia-induced sepsis. Nonetheless, HNF4α ligands and inhibitors remain at an experimental and developmental stage, limiting their current translation to the clinic. Further studies in clinically relevant models are therefore needed to determine whether HNF4α modulation may have therapeutic potential in specific forms of sepsis.

In conclusion, we demonstrate that hepatic metabolic suppression and HNF4α loss-of-function are conserved features of distinct sepsis models. Remarkably, however, hepatocyte-specific HNF4α deficiency confers profound protection during pneumonia-induced sepsis despite being detrimental in polymicrobial peritonitis [7]. The observed protection is independent of KCs but critically dependent on neutrophils and is accompanied by major alterations in hepatic metabolism. Together, these findings reveal a previously unrecognized liver-neutrophil axis linking hepatic metabolic state to antibacterial immunity and highlight the context-dependent consequences of HNF4α dysregulation during sepsis.

## 4. Materials and Methods

### 4.1. Mice

For the RNA-Seq experiment, eight-week-old male C57BL/6J mice were obtained from Janvier Labs (Le Genest-Saint-Isle, France). Genetically modified animals were bred in-house. HNF4α^fl/fl^ mice were originally generated by Dr. Frank Gonzalez (NIH, Bethesda, MD, USA) and are formally designated B6.129×1(FVB)-Hnf4a^tm1.1Gonz^/J [23]. Exons 4 and 5 of the *Hnf4a* gene were flanked by loxP sites using embryonic stem cell technology. The mice were backcrossed onto a C57BL/6J background and provided by Dr. Iannis Talianidis (University of Crete, Heraklion, Greece), courtesy of Dr. Frank Gonzalez, under a Material Transfer Agreement. HNF4α^fl/fl^ mice were subsequently crossed with Alb-CreERT2^Tg/+^ mice, kindly provided by Drs. D. Metzger and P. Chambon (IGBMC, Illkirch, France) [24]. The HNF4α^fl/fl^;Alb-CreERT2^Tg/+^ mice were originally described in [7]. For experiments involving KC depletion, HNF4α^fl/fl^;Alb-CreERT2^Tg/+^ were crossed with KC-DTR^Tg/+^ mice [16]. Experiments involving genetically modified mice were performed using age- and sex-matched littermate controls, aged 8–12 weeks. Genotype did not significantly affect body weight; therefore, body weight was not taken into account when administering the compounds. All mice received a fixed injection volume determined a priori. In total, 201 mice were used for this study.

Hepatocyte-specific HNF4α deletion was induced in HNF4α^fl/fl^;Alb-CreERT2^Tg/+^ mice and HNF4α^fl/fl^;Alb-CreERT2^+/+^ littermate controls by intraperitoneal administration of tamoxifen (1 mg per mouse) dissolved in a 1:8 ethanol:oil solution for five consecutive days. Complete depletion of hepatic HNF4α was confirmed three days after the final tamoxifen injection [7]. KC depletion was obtained through IP injection of 500 ng DT dissolved in PBS one day before infection. All control mice also received DT IP injection. To deplete macrophages, mice received 200 μL clodronate liposomes (Liposoma, Amsterdam, The Netherlands) by intravenous injection 24 h before *S. pneumoniae* infection. Control mice received an equivalent volume of control liposomes according to the same schedule. Neutrophils were depleted by intraperitoneal administration of an anti-Ly6G antibody (clone 1A8) or control antibody. Mice received 150 µg anti-Ly6G IP one day prior to *S. pneumoniae* infection, on the day of infection, and two days post-infection. This dosing regimen was used to maintain neutrophil depletion throughout the experimental period. Animals were allocated alternately to control and experimental treatment groups rather than based on cage location or another systematic characteristic. Experimental procedures and measurements were performed according to the same standardized protocols across all groups and by the same experimenter.

No formal a priori sample size calculation was performed. Initial experiments were conducted as pilot studies to assess variability, feasibility, and the biological effects of the interventions. Additional independent experiments were performed when appropriate to increase the number of biological replicates and to assess the reproducibility and robustness of the findings.

Group sizes could vary between experiments and genotypes due to differences in breeding outcomes and the availability of animals with the required genotype, as well as practical considerations such as differences in the available concentration of individual compounds or the experimental question. For example, in the experiment comparing clodronate liposomes with control liposomes in the KO genotype, the initial experimental design focused on determining whether clodronate treatment specifically abolished the protective phenotype observed in the KO animals. Therefore, a WT-clodronate group was not included in the initial experiment. In a subsequent independent repeat, a WT + clodronate group was added to assess the effect of clodronate treatment in WT animals. As this group was not part of the primary experimental question, it contained fewer animals than the main experimental groups. The final number of animals (n) per group is indicated in the figure and/or corresponding figure legends. Only healthy animals were included at the start of the experiments. No animals were subsequently excluded from the analyses.

Mice were housed in individually ventilated cages (IVCs) under standard housing conditions (22 °C; 14:10 h light/dark cycle) in a specific pathogen-free facility. Mice had ad libitum access to food (chow diet containing 18% protein, 4.5% fiber, 4.5% fat, and 6.3% ash; Provimi Kliba SA, Kaiseraugst, Switzerland) and water. All animal experiments were approved by the Institutional Ethics Committee for Animal Welfare of the Faculty of Sciences, Ghent University, Belgium and were performed in accordance to the ARRIVE 2.0 guidelines. 

### 4.2. Intratracheal S. pneumoniae Infection

In the current study, the bacterial *S. pneumoniae* strain TIGR4 was selected as the infectious agent because it is a clinical isolate that is also highly virulent in mice when administered intratracheally. TIGR4 was cultured in brain heart infusion (BHI) medium at 37 °C, 5% CO_2_, until an optical density of 0.3 was reached. Bacteria were subsequently collected by centrifugation at 3220× *g* for 5 min, and the bacterial pellet was resuspended in PBS and diluted to the required concentration. For intratracheal infection, mice were anesthetized with isoflurane and inoculated intratracheally with 2–5 × 10^5^ colony-forming units (CFU) of TIGR4 in 100 μL PBS. For tissue isolation experiments, mice were euthanized by cervical dislocation at the indicated experimental time points. In survival experiments, rectal body temperature was monitored daily. Mice were euthanized upon reaching predefined humane endpoints, defined as a rectal body temperature below 30 °C or a body weight loss exceeding 20% of baseline body weight.

### 4.3. RNA Isolation and RNA Sequencing

For RNA-Seq, livers of mice infected with *S. pneumoniae* or PBS were isolated two days after infection (n = 3/group). Liver tissue (bulk) was isolated and stored in RNAlater (Life Technologies Europe, Bleiswijk, The Netherlands). Total RNA was extracted using the Aurum Total RNA Mini Kit (Bio-Rad, Hercules, CA, USA) according to the manufacturer’s instructions. RNA concentration was determined using a NanoDrop 1000 spectrophotometer (Thermo Scientific, Wilmington, DE, USA), and RNA quality was assessed using a 5300 Fragment Analyzer (Agilent, Singapore, Singapore). RNA-sequencing libraries were prepared using the Illumina TruSeq Stranded mRNA Library Prep Kit (San Diego, CA, USA) at the VIB Nucleomics Core (Leuven, België) and sequenced on an Illumina NovaSeq 6000 device (San Diego, CA, USA). Single-end 100-bp reads were aligned to the mouse mm39 reference genome using STAR (v 2.7.11b) [25]. Differential gene expression analysis was performed using DESeq2 (DESeq2_1.50.2) [26], with a false discovery rate (FDR) of 5%. Gene set enrichment analysis was performed using Enrichr (October 17, 2025) [27] and HOMER 5.1. [28]. The identities of the genes shown in Figure 1C are provided in Dataset EV1.

For RT-qPCR, a total of 1 μg RNA was reverse-transcribed into cDNA using the iScript cDNA Synthesis Kit (Bio-Rad, Hercules, CA, USA). The resulting cDNA was diluted 1:10 in nuclease-free water prior to qPCR analysis. RT-qPCR was performed using a LightCycler 480 system (Roche, Rotkreuz, Switzerland) and SensiFAST SYBR No-ROX Mix (Bioline, London, UK). Samples were analyzed in technical duplicates. Target gene expression was normalized to the housekeeping genes *Gapdh* and *Hprt* (see Table 1 for primer sequences). qPCR data were analyzed using qbase+ software (Biogazelle, Ghent, Belgium).

### 4.4. Bacterial Burden

Bacterial loads in the spleen, liver, and lungs were determined by homogenizing each organ in 1 mL sterile PBS. A 20 μL aliquot of each homogenate was plated on blood agar plates. Colonies were counted 24 h after plating following incubation at 37 °C in 5% CO_2_, and bacterial burden was expressed as CFU/organ. For blood bacterial burden, 5 μL of blood collected from the tail was directly plated onto blood agar plates.

### 4.5. Biochemical Analysis

Plasma interleukin-6 (IL-6), free fatty acids (FFAs) and total cholesterol levels were measured using commercially available assay kits (eBioscience (88-7064-88, San Diego, CA, USA), Abnova (KA1667, Taipei, Taiwan) and Sigma (MAK043, St. Louis, MO, USA), respectively) according to the manufacturers’ instructions. Plasma was obtained from blood collected in EDTA-coated tubes by centrifugation at 2000× *g* for 15 min at 4 °C.

### 4.6. Phagocytosis Assay

*TIGR4* strain was cultured in BHI medium at 37 °C with 5% CO_2_ for 9 h. Bacteria were collected by centrifugation at 3220× *g* for 5 min, resuspended in 100 mM sodium bicarbonate (pH 8.5), and labeled with pHrodo Red dye (Thermo Scientific, Eugene, OR, USA) for 45 min at room temperature in the dark. Excess dye was removed by washing with HBSS. Bacteria were subsequently fixed in 100% methanol and resuspended in HBSS for intravenous administration. Following a 1.5-h period to allow in vivo phagocytosis, liver cells were isolated using a modified perfusion-digestion protocol. Briefly, livers were retrogradely cannulated and perfused for 2 min with EDTA-containing HBSS, followed by perfusion with 0.2 mg/mL Collagenase IV (Sigma-Aldrich, St. Louis, MO, USA) for 5 min at a flow rate of 6 mL/min. Excised livers were minced and further digested for 20 min at 37 °C in 0.4 mg/mL Collagenase IV and 10 U/mL DNase I (Roche, Mannheim, Germany). All subsequent procedures were performed at 4 °C.

The resulting cell suspension was filtered through a 100-µm mesh, subjected to osmotic erythrocyte lysis, and subsequently passed through a 40-µm filter. Hepatocytes and cellular debris were depleted by two sequential low-speed centrifugation steps at 50× *g* for 1 min. The non-parenchymal cell fraction was subsequently collected by centrifugation at 400× *g* for 5 min. For spectral flow cytometry, approximately 1–5 × 10^6^ cells were incubated with anti-CD16/32 (1:100; clone 2.4G2; Bioceros; AB_3746267, Utrecht, The Netherlands) to block Fc receptors and stained for 30–45 min at 4 °C. The antibody panel consisted of Fixable Viability Dye eFluor 780 (1:500; Thermo Fisher; 65-0865-14, Carlsbad, CA, USA), CD45-eFluor 506 (1:200; Thermo Fisher; AB_2637147, Carlsbad, CA, USA), CD31-eFluor 450 (1:500; Thermo Fisher; AB_10598807, Carlsbad, CA, USA), F4/80-BV785 (1:200; BioLegend; 123141, San Diego, CA, USA), CD64-AF647 (1:200; BioLegend; 305012, San Diego, CA, USA), CLEC2-PE-Cy7 (1:400; BioLegend; 146108, San Diego, CA, USA), and VSIG4-PE-eFluor 610 (1:400; Thermo Fisher; AB_2802395, Carlsbad, CA, USA). TIM-4-PE was omitted from the pHrodo phagocytosis panel because of spectral overlap with the pHrodo Red signal.

Data were acquired using a Cytek Aurora spectral flow cytometer and SpectroFlo software (Cytek Biosciences, Fremont, CA, USA). Flow cytometry data were analyzed using FlowJo v10.8 (BD Life Sciences, Ashland, OR, USA). Phagocytic activity was quantified as the mean fluorescence intensity (MFI) of the pHrodo Red signal within the live pHrodo+ CD45^+^CD31^−^F4/80^+^CLEC2^+^CD64^+^VSIG4^+^ KC population.

### 4.7. Statistical Analysis

Statistical analyses and data visualization were performed using GraphPad Prism 10.0 (GraphPad Software, Inc., Boston, MA, USA) or in R (v 4.5.0) for RNA-seq analyses. Comparisons between two groups were performed using an unpaired two-tailed Student’s *t*-test. For experiments involving two independent variables, two-way analysis of variance (ANOVA) was used. Survival distributions were analyzed using Kaplan–Meier curves and compared using the log-rank (Mantel–Cox) test. A *p*-value < 0.05 was considered statistically significant. Data are presented as individual data points, with each point representing an individual mouse, and bars representing the mean ± SEM, except for Figure 3D–F, where data from two experiments were pooled and analyzed using a generalized linear model (GLM) in RStudio. These plots show adjusted means ± SD. Statistical details are provided in the corresponding figure legends. “ns” indicates not significant. The experimenter was aware of group allocation during animal allocation, experimental procedures, and outcome assessment. Group allocation was masked during data analysis to minimize potential subjective bias in the statistical analysis and interpretation of the results.

## Figures and Tables

**Table 1:** Primer sequences used for RT-qPCR.

	Forward	Reverse
* Gapdh *	TGAAGCAGGCATCTGAGGG	CGAAGGTGGAAGAGTGGGAG
* Hprt *	AGTGTTGGATACAGGCCAGAC	CGTGATTCAAATCCCTGAAGT
* Clec4f *	GAGGCCGAGCTGAACAGAG	TGTGAAGCCACCACAAAAAGAG
* Albumin *	TGCTTTTTCCAGGGGTGTGTT	TTACTTCCTGCACTAATTTGGCA

## Data Availability

The original data presented in the study are openly available in the National Center for Biotechnology Information Gene Expression Omnibus (GEO) public database at http://www.ncbi.nlm.nih.gov/geo/ and are publicly available under accession number GSE345327.

## Supplementary Materials

The following supporting information can be downloaded at: https://www.mdpi.com/article/10.3390/ijms27188198/s1.

## Abbreviations

The following abbreviations are used in this manuscript:

ANOVA	Analysis of variance
bp	Base pairs
CFU	Colony-forming units
CLP	Cecal ligation and puncture
DT	Diphtheria toxin
DTR	Diphtheria toxin receptor
FFAs	Free fatty acids
GLM	Generalized linear model
HNF4a	Hepatocyte nuclear factor 4 alpha
IL-6	Interleukin-6
IT	Intratracheal
IV	Intravenously
iWAT	Inguinal white adipose tissue
KC	Kupffer cell
Liver-i-KO	Liver inducible knockout
LXR	Liver X Receptor
ns	Non-significant
Padj	Adjusted *p*-value
PBS	Phosphate-buffered saline
PPARα	Peroxisome proliferator–activated receptor alpha
RNA-seq	RNA-sequencing
RXR	Retinoid X Receptor
*S. pneumoniae*	*Streptococcus pneumoniae*
TF-LOF	Transcription factor loss-of-function
WAT	White adipose tissue
WT	Wild-type
